# Radiation-Inactivated *Acinetobacter baumannii* Vaccine Candidates

**DOI:** 10.3390/vaccines9020096

**Published:** 2021-01-27

**Authors:** Stephen J. Dollery, Daniel V. Zurawski, Elena K. Gaidamakova, Vera Y. Matrosova, John K. Tobin, Taralyn J. Wiggins, Ruth V. Bushnell, David A. MacLeod, Yonas A. Alamneh, Rania Abu-Taleb, Mariel G. Escatte, Heather N. Meeks, Michael J. Daly, Gregory J. Tobin

**Affiliations:** 1 Biological Mimetics, Inc., 124 Byte Drive, Frederick, MD 21702, USA; john.tobin@bmi-md.com (J.K.T.); wiggins@bmi-md.com (T.J.W.); bushnell@bmi-md.com (R.V.B.); dmacleod8@gmail.com (D.A.M.); tobin@bmi-md.com (G.J.T.); 2 Wound Infections Department, Bacterial Diseases Branch, Center for Infectious Disease Research, Walter Reed Army Institute of Research, Silver Spring, MD 20910, USA; dvzurawski@gmail.com (D.V.Z.); yoann.s.lebreton.ctr@mail.mil (Y.A.A.); rania.abu-taleb.ctr@mail.mil (R.A.-T.); mariel.g.escatte.ctr@mail.mil (M.G.E.); 3 Department of Pathology, Uniformed Services University of the Health Sciences, Bethesda, MD 20814, USA; elena.gaidamakova.ctr@usuhs.edu (E.K.G.); vera.matrosova.ctr@usuhs.edu (V.Y.M.); michael.daly@usuhs.edu (M.J.D.); 4 Henry M. Jackson Foundation for the Advancement of Military Medicine, Bethesda, MD 20817, USA; 5 Defense Threat Reduction Agency, Ft. Belvoir, VA 22060, USA; heather.n.meeks4.civ@mail.mil

**Keywords:** *A. baumannii*, vaccine, mouse, whole-cell, irradiated, protective, MDP, inactivated, pulmonary, *Deinococcus*

## Abstract

*Acinetobacter baumannii* is a bacterial pathogen that is often multidrug-resistant (MDR) and causes a range of life-threatening illnesses, including pneumonia, septicemia, and wound infections. Some antibiotic treatments can reduce mortality if dosed early enough before an infection progresses, but there are few other treatment options when it comes to MDR-infection. Although several prophylactic strategies have been assessed, no vaccine candidates have advanced to clinical trials or have been approved. Herein, we rapidly produced protective whole-cell immunogens from planktonic and biofilm-like cultures of *A. baumannii*, strain AB5075 grown using a variety of methods. After selecting a panel of five cultures based on distinct protein profiles, replicative activity was extinguished by exposure to 10 kGy gamma radiation in the presence of a *Deinococcus* antioxidant complex composed of manganous (Mn^2+^) ions, a decapeptide, and orthophosphate. Mn^2+^ antioxidants prevent hydroxylation and carbonylation of irradiated proteins, but do not protect nucleic acids, yielding replication-deficient immunogenic *A. baumannii* vaccine candidates. Mice were immunized and boosted twice with 1.0 × 10^7^ irradiated bacterial cells and then challenged intranasally with AB5075 using two mouse models. Planktonic cultures grown for 16 h in rich media and biofilm cultures grown in static cultures underneath minimal (M9) media stimulated immunity that led to 80–100% protection.

## 1. Introduction

The increase in multidrug-resistant (MDR) bacteria is a burgeoning concern. The World Health Organization (WHO) lists the Gram-negative *Acinetobacter baumannii* in their highest category of pathogens posing an imminent threat to human health based upon factors including how deadly infections are, how frequently cases occur, the available treatment options, and the ease of containment for a given pathogen [1]. The CDC list carbapenem-resistant *Acinetobacter* first in the 2019 Antibiotic Resistance Threats in the United States report [2]. *A. baumannii* encodes numerous antibiotic resistance mechanisms, including β-lactamases, efflux pumps, aminoglycoside-modifying enzymes, permeability defects, mechanisms to alter antibiotic target sites [3], and gene amplification systems that lead to transient MDR-phenotypes [4]. The bacteria are also known to have a high natural competence, incorporating exogenous DNA at high frequencies and making resistance to future antibiotics a real prospect [5,6,7]. In the United States, there are approximately 45,000 infections per year and around one million globally [8]. Infections are twice as prevalent in Asia and the Middle East, and *A. baumannii* is the predominant nosocomial pathogen in some countries [9]. Mortality rates between 15% and 90% are frequently cited depending on strain and setting [10,11].

*Acinetobacter* spp. represent one of the most environmentally robust groups of organisms identified, surviving extreme exposures to desiccation. Following an evolutionary shift that promotes survival in the human host, *A. baumannii* infections are now predominantly nosocomial. However, *A. baumannii* retained some of the environmental predecessor’s traits and can survive on the surfaces of hospital facilities and equipment for weeks as desiccated biofilms and persist through many commonly used decontamination procedures [12,13] because it has retained many of these extreme survival characteristics.

Reports show that treatment with minocycline can reduce the mortality from approximately 50% to 20% if treatment starts promptly [14]. However, many strains are resistant to tetracyclines and even newly approved antibiotics such as omadacycline and eravacycline [15]. Therapeutic antibodies are in development, but those targeting polysaccharides might not provide cross-strain protection. For example, a recent study showed that a promising monoclonal antibody against capsule did not bind to 39% of tested strains [16]. Alternative therapies such as lysogenic phage have also shown promise [17] but require more clinical research before effective therapies can be made available. In contrast, the protection from a strong and directed immune response would target multiple bacterial antigens using multiple host attack mechanisms from both arms of the immune system. A broadly protective immune response to whole-cell bacteria, as compared to a narrowly focused immunity against a single subunit protein, could reduce the evolution of escape variants. For these reasons, vaccines are considered primary solutions to the antibiotic-resistance problem, and herd immunity may even reverse the prevalence of MDR strains [18,19,20]. Unfortunately, no effective *A. baumannii* vaccine candidates have entered clinical trials, and a vaccine is urgently needed.

Historically, the discovery and commercial development of a licensed vaccine has taken decades of basic research followed by years of pre-clinical and clinical development as well as regulatory approval. Several recent outbreaks, epidemics, and pandemics caused by emerging and reemerging pathogens (e.g., *M. tuberculosis,* *B. anthracis, Y. pestis,* Zika virus, Venezuelan equine encephalitis virus, pandemic influenza, and Coronaviruses) underscore the importance of developing vaccines much more rapidly. One whole organism vaccine development strategy consists of chemically inactivating the pathogen and has been successfully used against a variety of important pathogens (e.g., poliovirus, influenza, cholera, plague, pertussis, and typhoid). We have developed an improved method for generating inactivated vaccines from whole pathogens that is rapid, scalable, and inexpensive [21,22,23].

Exposure to ionizing forms of radiation (X-rays and gamma rays) is commonly used for sterilizing laboratory and medical supplies and has been reported as a strategy for vaccine production since the early days of vaccinology [24,25,26]. The sterilizing effects of ionizing radiation are ascribed to the sum of two destructive processes. Under aqueous conditions, proteins and nucleic acids of a pathogen are damaged indiscriminately by photons, but more selectively and severely by the indirect action of reactive oxygen species (ROS) generated from the radiolysis of water molecules [27,28]. For vaccine production, radiation-induced destruction of the nucleic acids inside the pathogen is desired while damage to the structural proteins on the outside of the pathogen is not because it reduces antigenic potency. Using a newly developed technology, proteins can be specifically protected from the indirect effects of gamma radiation, leaving DNA and RNA genomes open to destruction [21,22,23].

The extremotolerant bacterium *Deinococcus radiodurans* is exceptionally resistant to gamma radiation and can survive doses of 12–16 kGy [29,30,31,32]. In comparison, humans cannot survive 10 Gy and most bacteria are killed by less than 1 kGy [31,33]. Dr. Daly and colleagues initially discovered that the key to the extraordinary radioresistance of *D*. *radiodurans* is proteome protection, which is mediated by the accumulation of small-molecule manganese (Mn)-antioxidants that prevent protein hydroxylation and carbonylation during irradiation by scavenging superoxide [28,32].

In *D. radiodurans*, Mn-antioxidants make up approximately 70% of the cytosolic Mn^2+^, forming complexes with orthophosphate (Pi) and peptides [30,31]. Through the analysis of naturally-occurring *Deinococcus* Mn complexes, the rational design of the peptide components of Mn antioxidants has yielded a synthetic complex named MDP (Manganese-Decapeptide-Phosphate) which forms spontaneously when the decapeptide DEHGTAVMLK, Pi, and Mn^2+^ are combined [21,30,34]. Numerous studies have elucidated the ROS-scavenging mechanisms of MDP along with proof-of-concept and vaccine development studies in which the antigenic structures of poliovirus, bacteriophage Lambda, Venezuelan equine encephalitis virus (VEEV), Chikungunya virus, and *Staphylococcus aureus* (MRSA) were protected by MDP during supra-lethal exposure to gamma rays [21,22,23,34].

Herein, we have extended this approach to the development of *A. baumannii* immunogens and vaccine candidates. To generate the most protective vaccines, we sought to generate *A. baumannii* cultures using a variety of different growth conditions to ensure the production of a variety of different surface proteins and epitopes. *A. baumannii* cultures that grow in different environments have different protein expression profiles as they adapt for optimal growth [35]. We selected five culture conditions that yield different protein profiles, and gamma-inactivated these cultures in the presence of the epitope-protecting MDP complex to generate whole-cell vaccine candidates. Upon vaccination and challenge in two animal models, we observed unsurpassed efficacy for several candidates but Is the bold necessary? The following highlight are the samenot others.

## 2. Materials and Methods

### 2.1. Materials

Materials were purchased from Sigma Chemical Company, St. Louis, MO, US, if not stated otherwise. Bacto-tryptone, yeast extract, bacto-agar, trypticase soy agar (TSAII), and casamino acids were from BD, Franklin Lakes, NJ, USA. Trypticase soy agar plates with 5% sheep blood were from Thermo Scientific, Dubuque, IA, USA. Tryptic Soy Broth (TSB) was from General Laboratory Products, Yorkville, IL, USA. The 10× PBS (Phosphate Buffered Saline) pH 7.4 was from Quality Biological, Inc., Gaithersburg, MD, USA.

### 2.2. Bacterial Growth Conditions

All work was carried out under Biosafety Level II conditions.

Overnight cultures in TSB medium (planktonic #1, P1). AB5075 pre-grown during the day in 5 mL of TSB was diluted up to (Optical Density) OD_600_ = 0.05 in 25 mL TSB and incubated overnight (~16 h) at 37 °C, 200 rpm until OD_600_ = 7. Resulting cultures were concentrated 5-fold for all downstream analyses. 

Two-hour cultures in TSB medium (planktonic #2. P2). Overnight cultures of AB5075 were diluted to OD_600_ = 0.05 in 60 mL TSB and incubated at 37 °C, 200 rpm for about 2 h in TSB until OD_600_ = 0.7. Resulting cultures were concentrated 50-fold for all downstream analyses.

Submerged biofilm cultures (biofilm #1. B1). *A. baumannii* AB5075 was grown overnight at 37 °C on M9 agar plates. Liquid cultures of AB5075 were grown in M9 minimal medium supplemented with 2 mM MgSO_4_, 0.4% glucose, 0.1 mM CaCl_2_, and 0.1% casamino acids. This medium is referred to throughout the text as “M9 medium” [36]. The next day, 65 mL of M9 minimal medium was inoculated with AB5075 from a freshly streaked plate and incubated overnight at 37 °C while shaking at 200 rpm. The following day, the overnight culture was diluted in M9 media to an OD600 of 0.05. The final volume in each 225 cm^2^ flask was 100 mL. Up to ten flasks were inoculated for each biofilm vaccine candidate preparation for mouse experiments. Media and culture were mixed and placed in a 37 °C incubator without shaking. Media was replaced with fresh M9 media on day 3. On day 4, media and floating cells were removed from the flask and replaced with 25 mL of 4 °C cold PBS. Cells were scraped into the cold PBS from the bottom of the flask using a plastic cell scraper and collected.

Biofilm on the surface of M9 agar (biofilm #2, B2) and on the surface of TSAII + 5% sheep blood plates (biofilm #3, B3). AB5075 was grown as a lawn on the surface of the plates (overnight) at 37 °C. The next day the cells were collected from the surfaces of the plates using a soft plastic scraper and washed in cold PBS.

### 2.3. Formulation of Vaccine Candidates in MDP Prior to Irradiation

The synthetic decapeptide (DP1) H-Asp-Glu-His-Gly-Thr-Ala-Val-Met-Leu-Lys-OH [28] was custom-synthesized and refined to 95% purity by Sigma-Aldrich, The Woodlands, TX, USA, and concentrations of stock DP1 solutions were confirmed by LC-MS. Before irradiation, AB5075 cells were washed twice with ice-cold PBS pH 7.4 and resuspended in PBS and adjusted to final concentrations of 1 mM MnCl_2_, 3 mM DP1, and 25 mM potassium phosphate buffer, pH 7.4 (MDP), using stock solutions. Stock solutions of 100 mM MnCl_2_ (Sigma), 30 mM DP1, and 1 M potassium phosphate buffer (pH 7.4) were prepared in ultrapure water from a Barnstead Nanopure Diamond ultrapure water purification system. Cell concentrations for irradiation were between 10^10^ and 10^11^ colony-forming units (CFU)/mL.

### 2.4. Cobalt-60 Irradiations and Inactivation Curves

Irradiations were performed on wet ice with a ^60^Co source emitting between 7 and 16.4 kGy/h. For inactivation curves, samples were washed in PBS pH 7.4 and irradiated with or without MDP at increasing doses of gamma irradiation. Following irradiation, the bacteria were serially diluted (10^−1^ to 10^−8^) in cold PBS and plated onto TSAII plates. Following an overnight incubation at 37 °C, the number of colonies were enumerated and the CFU/mL calculated from the dilution factors. For mouse experiments, samples of AB5075 at 1.0 × 10^10^ CFU per ml were irradiated with 10 kGy to ensure a lack of CFU. Following irradiation, approximately 1,000 immunization doses of AB5075 (1.0 × 10^10^ CFU) were cultured on TSAII plates to ensure that no residual replicative activity remained. Immunogens were stored at 4 °C until immunization.

### 2.5. Stability Assays

Stability assays were conducted on bacteria with or without MDP that were either not irradiated or irradiated with 10 kGy and stored at 4 °C. At varying time points, the non-irradiated samples were analyzed for viability of the bacteria by plating serial dilutions of the sample onto TSAII agar plates to determine CFU/mL. The irradiation-killed samples were analyzed during the same time course by counting under a microscope using a hemocytometer.

### 2.6. Coomassie-Stained Protein Gels

For Coomassie-stained protein gel analysis, 50 µL volumes containing 5.0 × 10^8^ CFU of bacteria from the five samples were mixed with equal volumes of 2× Laemmli SDS-PAGE sample buffer (Bio-Rad, Hercules, CA, USA). The samples were vortexed, heated for 15 min at 100 °C, vortexed again, and centrifuged at 10,000× *g* for 5 min to clarify the crude lysates. The samples were electrophoresed in denaturing polyacrylamide gels (4–20% acrylamide). After electrophoresis, the gels were stained with Bio-Safe Coomassie G-250 stain (BioRad, 161-0786) and images were acquired using Odyssey CLx imaging system (LI-COR Biosciences, Inc, Lincoln, NE, USA).

### 2.7. Two-Dimensional Electrophoresis

The P1 and B1 (described in Table 1) cultures were analyzed for protein profiles using 2D electrophoresis. Approximately 2 × 10^9^ CFU of each culture were mixed in 100 µL of denaturing SDS-PAGE sample buffer and boiled for 5 min to disrupt the bacterial cells. The samples were centrifuged at 18,000× *g* for 20 min and the clarified extracts transferred to fresh tubes. 2D electrophoresis was performed according to the carrier ampholine method of isoelectric focusing by Kendrick Labs, Inc. (Madison, WI, USA). Briefly, isoelectric focusing was carried out in a glass tube of inner diameter 3.3 mm using 2.0% pH 3–10 Isodalt Servalytes (Serva, Heidelberg, Germany) for 20,000 volt-h. One µg of an IEF internal standard, tropomyosin, was added to each sample. After equilibration for 10 min in buffer “O” (10% glycerol, 50 mM dithiothreitol, 2.3% SDS and 0.0625 M tris, pH 6.8), each tube gel was sealed to the top of a stacking gel that overlaid a 10% acrylamide slab gel (1.0 mm thick). SDS slab gel electrophoresis was carried out for about 5 h at 25 mA/gel. After electrophoresis, the gels were either stained with Coomassie R-250 and photographed or transferred to PVDF membrane by electroblotting and processed for Western blotting. The following proteins (MilliporeSigma, MA, USA) were used as molecular weight standards: myosin (220,000), phosphorylase A (94,000), catalase (60,000), actin (43,000), carbonic anhydrase (29,000), and lysozyme (14,000). These standards appear as bands at the basic edge of the stained gels and Western blots. For Western blotting, the membranes were blocked for 2 h in 5% nonfat dried milk solubilized in PBS with 0.1% Tween 20 (PBS-T), reacted with a 1:2500 dilution of sera from rats immunized with *A. baumannii* AB5075 (P1), washed, and probed with HPR-conjugated goat anti-rat sera. Proteins that appear specific to one of the two cultures are highlighted with arrows. To generate anti-AB5075 planktonic and biofilm rat sera, Wistar rats were immunized with inactivated cultures grown as P1 (as described above) or B1 under LB media. The rats received 1.0 × 10^7^ cells on days 1, 21, and 42. Animals were euthanized and bled out on day 54.

### 2.8. Challenge in the Murine Pulmonary Models

All mouse studies were conducted in accordance with the Guide for the Care and Use of Laboratory Animals (National Research Council, 2011), and procedures were approved by the Institutional Animal Care and Use Committee at the Walter Reed Army Institute of Research (protocol 16-BRD-48S). The murine pulmonary model was used as previously described [37]; however, animals were vaccinated with inoculum prepared under each irradiated-growth condition at sixty days before challenge. A total of 100 µL of vaccine candidate (1.0 × 107 irradiated bacterial cells) was injected intramuscularly (IM) into one quadriceps of each mouse. Four weeks and six weeks later, a booster was provided IM in each animal, same as before. At eight weeks post-vaccination and two weeks from last booster, the animals were challenged with an intranasal inoculum of AB5075. Two sets of experiments were done. One set of mice, BALB/c mice, were given cyclophosphamide on days 4 and 1 before an intranasal challenge of 5.0 × 10^6^ CFU in 50 µL of sterile saline (LD80). Another set of mice, C57BL/6 mice, were not treated with cyclophosphamide, and instead just challenged with an intranasal dose of 1.0 × 10^8^ CFU in 50 µL, similar to a previous report (LD50) [38]. In both sets of experiments, mice were observed for one week, and each experiment was repeated at least once (*n* = 20 mice). Animals were monitored for morbidity and mortality for 7 days, and humanely euthanized with CO_2_ inhalation when clinical scoring suggested succumbing to infection.

## 3. Results

### 3.1. Growth of A. baumannii Cultures with Differing Protein Expression Profiles

*A. baumannii* cultures that grow in different environments have different protein expression profiles as they adapt for optimal growth [35]. Deriving conditions that express the most protective protein, or combination of proteins may prove pivotal in generating an effective vaccine. As our primary goal was to generate a whole-cell irradiated vaccine, we sought to prepare and test several whole-cell candidate vaccines with unique protein expression profiles. Based on prior observations, we grew *A. baumannii* under 5 distinct culture conditions as detailed in Table 1. Two planktonic cultures were grown in shaking flasks containing tryptic soy broth (TSB) and were harvested at two time points (culture P1, overnight to stationary growth phase; culture P2, at two hours to logarithmic growth phase). Three biofilm cultures were also grown. Culture B1 incubated under M9 media for two days adhered to the inner surface of a tissue culture flask, and biofilm cultures B2 and B3 were grown overnight on minimal medium (M9) or trypticase soy agar (TSA) supplemented with sheep red blood (SB), respectively.

**Figure 1:**
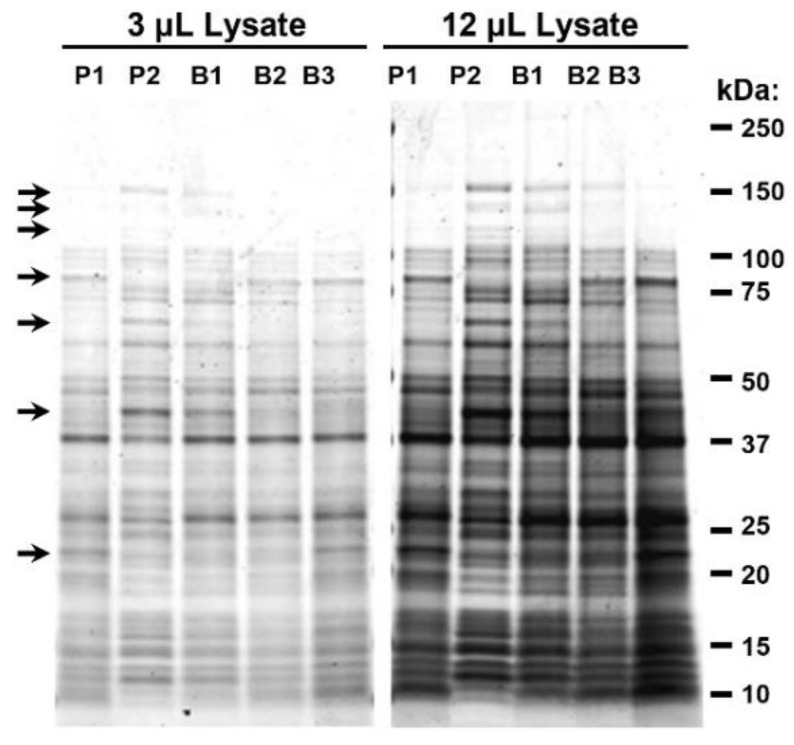
Analysis of proteins from *A. baumannii* cultures grown under varying conditions.

To validate that the growth conditions resulted in different protein profiles, we performed SDS-PAGE analysis of the five *A. baumannii* cultures. As had been seen in preliminary studies, each culture displayed a unique and distinct protein profile. The relative ratios of many proteins were observed to be different between samples with some proteins appearing entirely unique to a particular culture. Selected unique bands at various electrophoretic mobilities are indicated with arrows (Figure 1). Table 2 summarizes select differences in protein profiles observed by Coomassie stain.

Samples of P1, P2, B1, B2, and B3 were cultured, harvested, denatured, resolved via SDS-PAGE, and visualized by Coomassie Blue staining. The electrophoretic mobilities of molecular weight markers are indicated. Arrows highlight bands where obvious differences can be seen between samples. Sample loading volumes of 3 and 12 µL are shown to highlight differences between high and low abundance bands.

### 3.2. Antigenic Analysis of A. baumannii Cultures

**Figure 2:**
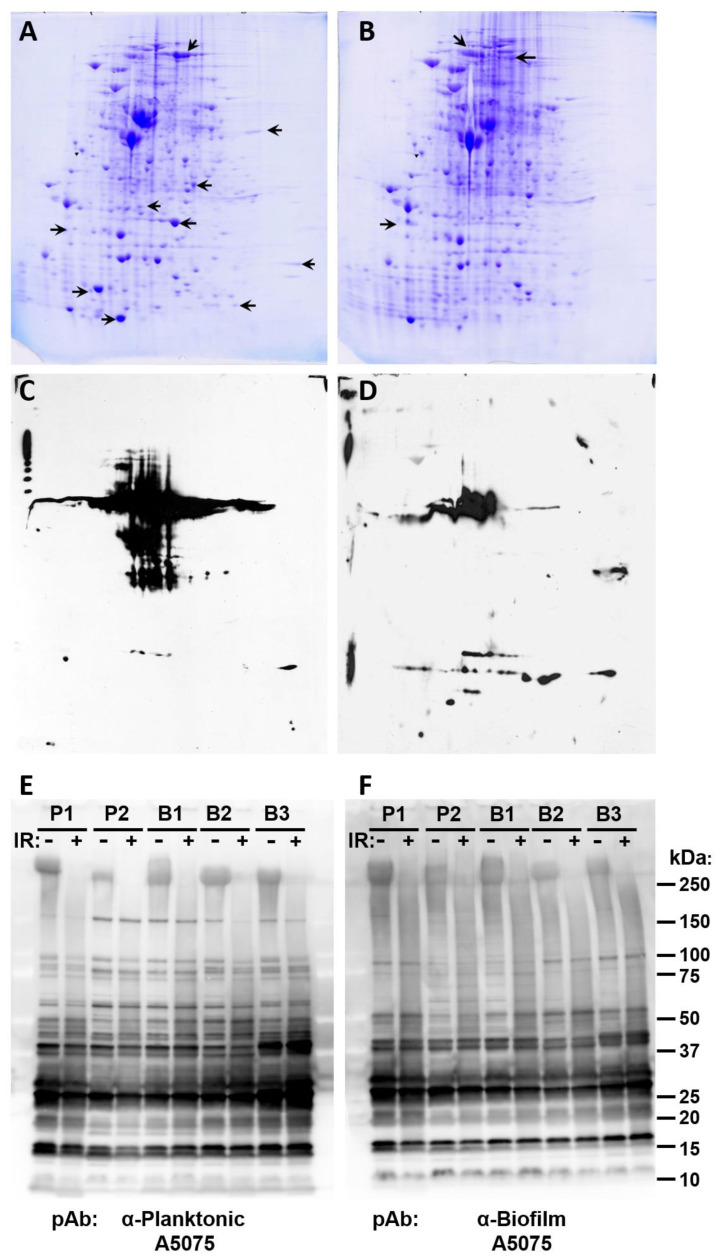
Denaturing gel analysis of proteins from planktonic and biofilm cultures of *A. baumannii.* Two-dimensional electrophoresis gels are shown of cell lysates from cultures harvested in (**A**,**C**) planktonic growth phase (P1) or in (**B**,**D**) biofilm phase (B1). The gels in Panels A and B were stained with Coomassie Blue to show all visible proteins. Arrows point to several proteins that are present or increased in that gel, but that are absent or decreased in the accompanying gel. Replicate gels were transferred to membranes and probed with polyclonal rat antisera raised against *A. baumannii* (panels C and D). Panel C shows proteins detected from planktonic growth phase (P1) and Panel D shows proteins detected from biofilm phase (B1). Panels E and F show Western blot analysis of P1, P2, B1, B2, and B3 lysates before and after irradiation in the presence of MDP probed with polyclonal rat sera raised against either planktonic (**E**) or biofilm (**F**) forms of *A. baumannii* AB5075.

To further analyze the potential differences in protein composition between planktonic and biofilm forms of *A. baumannii*, 2D electrophoresis followed by Coomassie staining (Figure 2A,B) and 2D Western blots (Figure 2C,D) were performed comparing P1 and B1. As expected, 2D Coomassie staining revealed that many additional differences in protein expression can be seen between samples using this method. Again, the relative ratios of many proteins were observed to vary greatly between samples with some proteins appearing entirely unique. Further differences between samples are shown in Figure 2C,D when proteins were detected using sera from a rat immunized with inactivated P1 planktonic cells.

To further examine the antigenic nature of planktonic and biofilm forms of *A. baumannii* and simultaneously test the stability of epitopes during irradiation, Western analysis was performed on un-irradiated immunogen samples (+MDP) and samples irradiated in the presence of MDP. Replicate blots were then probed with either polyclonal antibody raised against planktonic or biofilm *A. baumannii*. As expected, antibodies against biofilm or planktonic cultures detected numerous proteins in both the biofilm and planktonic cultures (Figure 2E,F). Many differences were observed between planktonic and biofilm sera, indicating variable immune responses in animals challenged with the different cultures. In addition, it appears that there is minimal epitope damage resulting from irradiation and that the epitopes in the cultures appear generally unaltered following irradiation with the exception of one higher molecular weight band.

Altogether, these results indicate that the cultures that we selected have different protein profiles, are antigenically distinct, and may be capable of inducing different protective immune responses following vaccination. Additional work will be needed to exhaustively examine the proteomic and immunogenic profiles of these cultures.

### 3.3. Gamma Irradiation Inactivates Replication of Planktonic and Biofilm Cultures of A. baumannii in the Presence or Absence of the Protective MDP Complex

**Figure 3:**
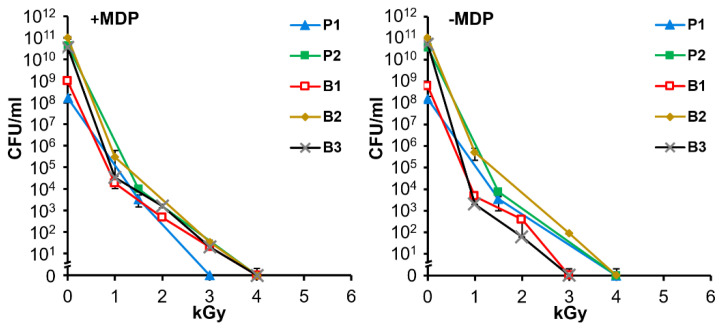
Irradiation inactivation of *A. baumannii* colony-forming units. *A. baumannii* samples P1, P2, B1, B2, and B3 were irradiated with or without MDP (as indicated) and analyzed for replicative viability. Serial dilutions of irradiated bacteria were plated to determine CFU/mL.

To determine appropriate inactivation and vaccine preparation procedures for *A. baumannii*, cultures were subjected to a range of doses of gamma irradiation in the presence and absence of MDP. Figure 3 shows the susceptibility of five bacterial preparations to gamma-irradiation. As previously observed in studies with MRSA [21], the presence of the ROS-scavenging complex, MDP, has minimal protective effect against lethal damage. In multiple experiments in which >1.0 × 10^10^ CFU of bacteria were irradiated with 10 kGy, no residual CFU were detected and this dose was selected for preparation of immunogens. 

### 3.4. Irradiated and Non-Irradiated A. baumannii Cultures Display Stability for at Least 4 Months at 4 °C

**Figure 4:**
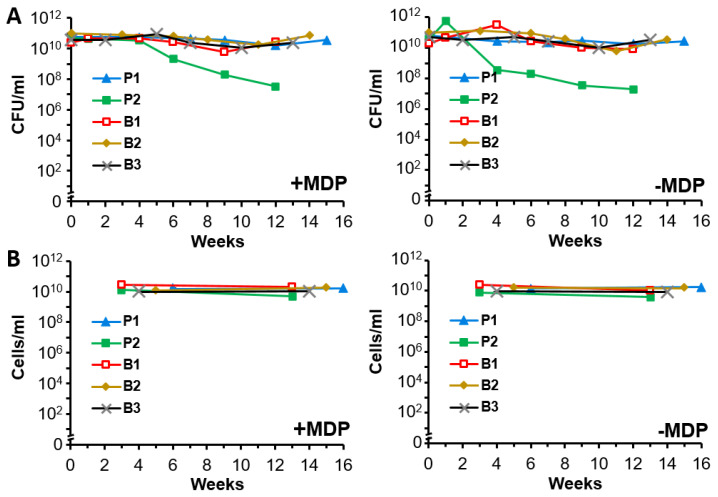
Stability of unirradiated and irradiated bacterial cultures. (**A**) Stability of unirradiated *A. baumannii* at 4 °C. P1, P2, B1, B2, and B3 cultures were prepared with or without the MDP complex and stored at 4 °C for up to 16 weeks at concentrations between 10^10^ and 10^11^ CFU/mL. A portion of each sample was removed for colony counting by plating serial dilutions on agar plates at the indicated time points. (**B**) Stability of irradiated *A. baumannii* at 4 °C. P1, P2, B1, B2, and B3 cultures were prepared with or without the MDP complex, exposed to 10 kGy gamma irradiation, and stored at 4 °C for up to 16 weeks at concentrations between 10^10^ and 10^11^ CFU/mL. A portion of each sample was removed and serially diluted for microscopic examination. The concentration of intact cells/mL are reported at the indicated time points.

Preliminary stability assays were conducted to determine the long-term viability of the bacteria and monitor the morphological structure of irradiated vaccine preparations at 4 °C. Samples prepared with or without MDP were either not irradiated (Figure 4A) or were irradiated with 10 kGy (Figure 4B) and stored at 4 °C. At varying time points, the non-irradiated samples were analyzed for viability by plating serial dilutions of the sample onto agar plates and counting CFU to quantitate viable cells per mL. With the exception of the samples from logarithmic planktonic growth conditions (P2), the various preparations maintained their starting viability for at least 12 weeks (Figure 4A). The irradiation-killed samples were analyzed during the same time period by counting whole intact bacteria microscopically as a method of detecting gross degradation from lysis. Minimal differences were seen over time (Figure 4B) indicating that vaccine preparations maintain their gross structure at 4 °C for up to 14 weeks. It should be noted that fresh preparations of immunogens were used in the following vaccination studies. The efficacy of stored immunogens will be examined in future experiments.

### 3.5. Irradiated A. baumannii Vaccines Are Highly Protective in Healthy Mice

**Figure 5:**
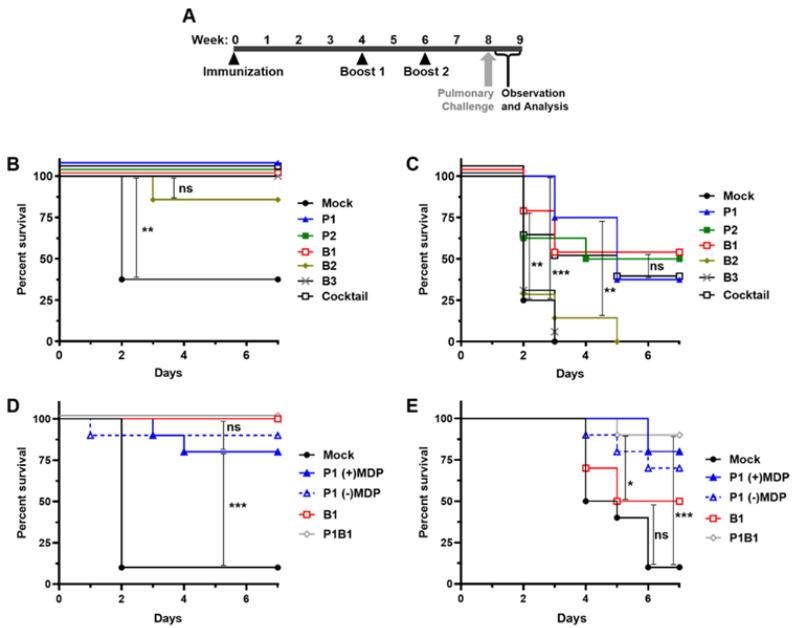
Immunization and intranasal challenge of mice with *A. baumannii*. (**A**) Graphic representation of the vaccination schedule. Mice were immunized at weeks 0, 4, and 6. Mice were challenged intranasally at week 8 and monitored daily until the end of week 9. (**B**–**E**) Kaplan–Meier Survival Analysis of vaccinated, or mock vaccinated mice challenged with AB5075 via intranasal inoculation as detailed in A. Panels B and C show groups of 8 mice immunized with candidate vaccines as indicated. B Shows survival in a healthy C57BL/6 mouse model, C shows survival in a neutropenic BALB/c model. Panels D and E show a second round of experiments using groups of 10 mice in the healthy C57BL/6 and neutropenic BALB/c models respectively. Log-rank (Mantle–Cox) test results of significance between survival numbers of groups is indicated by ns (not significant, *p* > 0.05), * *p* < 0.05, ** *p* < 0.01, and *** *p* < 0.001.

To test the protective effect of candidate *A. baumannii* vaccines, C57BL/6 mice were immunized with 1.0 × 10^7^ inactivated CFU-equivalents of P1, P2, B1, B2, B3, and a combination of all five (Cocktail), according to the vaccination schedule outlined in Figure 5A. C57BL/6 mice were challenged with a previously established LD50 of AB5075 via intranasal delivery and observed for 7 days. A protective effect on survival was observed in all of the mice that received candidate vaccines (Figure 5B). One mouse inoculated with the B2 vaccine was not protected, indicating that the B2 vaccine may not induce protective immunity as well as the other vaccine candidates. In comparison, 5 of 8 mice vaccinated with no antigen were not protected, as would be expected in this model. The Kaplan–Meier estimate was used to measure survival over time and the significance of differences between groups was analyzed using the Log-rank (Mantle–Cox) test. Protection from vaccination was determined to be significant for each group in comparison to mock-vaccinated mice.

To better understand differences in the protective effects elicited by immunogens P1, P2, B1, B2, B3, and the cocktail of all five immunogens, we tested the candidates in a neutropenic mouse challenge model of infection. Cyclophosphamide-treated BALB/c mice were given the same vaccination regimen and challenged similarly via pulmonary exposure (Figure 5C). The cyclophosphamide-treated mouse model revealed greater differences in protection between groups as circulating immune cells were not present to help clear the infection. Some differences were statistically significant. 100% of the mock vaccinated mice were not protected. The groups inoculated with B2 and B3 were also not protected with some mice surviving two days longer indicating a minimal protective response in B2. In all other groups, a protective effect was seen in around 50% of the mice. Interestingly, the mice in group P1 showed the highest levels of initial protection but then the levels dropped to slightly lower than several other groups at later time points. However, overall protection in P1 was not statistically different to the other samples affording the most protection. Although similarly efficacious, the combination of immunogens used in the cocktail did not result in enhanced protection.

### 3.6. Select A. baumannii Vaccines Are Highly Protective in Neutropenic Mice

In a second set of experiments using both mouse models, immunogens were down-selected to two of the most protective preparations, P1, B1, the combination of P1 and B1, and P1 prepared in the absence of MDP. Due to limitations on study size, other combinations were not tested. In healthy C57BL/6 mice, all three immunogens protected all or most of the mice (Figure 5D). In the neutropenic BALB/c mice, the combination of P1and B1 protected nine of ten mice, P1 alone with MDP protected eight, and B1 alone protected five (Figure 5E). In both experiments, the combination of P1 and B1 together performed the best with 9 out of 10 mice surviving in the neutropenic model. In this experiment B1 was seen to be as protective as in the prior experiment however P1 and P1B1 were again seen to be very protective (P1B1 vs. Mock = *p* 0.0004), with no significant difference seen between P1 and P1B1 (*p* > 0.05). Interestingly, under these conditions, no statistical difference was seen in samples prepared with or without the MDP complex.

## 4. Discussion

We previously demonstrated the use of MDP to protect protein epitopes of MRSA inactivated by 25 kGy [21]. The irradiated MRSA vaccine elicited both antibody and Th17 responses, and induced B and T cell-dependent protection in a mouse skin wound model. Structural integrity of the MRSA bacteria was shown to be preserved by MDP at gamma radiation doses far above those which abolish infectivity. Indeed, a dose of 25 kGy exceeds the outer limits of survival for all known bacteria under aqueous conditions [27,31]. For radiation-sensitive organisms, sterilization doses could be reduced significantly, which would be expected to reduce damage to epitopes needed to develop a protective immune response while maintaining an adequate margin of safety to ensure complete inactivation. Here we chose a gamma radiation dose of 10 kGy that yielded no surviving MDP-treated *A. baumannii* cells, starting with AB5075 cultures of 1.0 × 10^10^ CFU/mL which is estimated to be at least 1000 immunization doses (Figure 3).

The establishment of optimal conditions for propagating cells is essential in the production of whole-cell bacterial vaccines to ensure that they express proteins and other antigens that stimulate robust immunity. After assessing protein expression profiles for *A. baumannii* grown under more than a dozen conditions, we selected five different culture conditions that yielded distinct protein expression profiles (Figure 1). The panel included two planktonic and three biofilm cultures. P1 and P2 were grown in rich medium (TSB) to stationary and exponential planktonic growth phases, respectively. B1 was grown as a biofilm on the surface of tissue culture flasks beneath minimal M9 medium. B2 and B3 were grown as colony biofilms in air on M9 or on rich (TSA) medium supplemented with red blood cells, respectively. In addition, a sixth culture (cocktail) was composed of an equal quantity of each of the five cell preparations. The bacteria were complexed with MDP, inactivated with 10 kGy, and tested for immunogenicity in two mouse models of pulmonary infection. Immune-competent C57BL/6 mice are susceptible to infection by *A. baumannii* using the pulmonary route. BALB/c mice are less susceptible and pre-treatment with cyclophosphamide to reduce neutrophil activity is required to increase sensitivity to infection and reproducibility of disease with AB5075. In these studies, the two lead candidates were the planktonic cells grown to stationary phase and the biofilm grown under M9 media, which were stable at 4 °C for up to 16 weeks (Figure 4). From a pathogenesis perspective, this is an intriguing result. While we know the in vivo lifestyle of the bacteria must include a counter-response to the stress of the host environment, it is not clear if biofilms play a substantial role of pulmonary infection with regard to *A. baumannii*. Certainly, biofilms have been observed in wound infection [39,40] and urinary tract infections [41], but a role in pulmonary infection is less clear [42]. In vitro, there are numerous descriptions of interactions between *A*. *baumannii* and lung cells in tissue culture adherence assays and there are clearly relationships between adherence, biofilm, and iron acquisition activities [43,44,45]. In vivo, we have observed droplet-like structures in interstitial space between cells in the lung that have an ordered appearance in standard histopathology staining suggesting biofilm-like assembly; however, it is not clear how similar or different these structures are when compared to a biofilm formed in vitro or in a wound bed. A recent study implicated some of the same adherence and iron acquisition genes involved in biofilm formation and pulmonary infection. Another study also showed the importance of the Type II secretion system (T2SS) and a role for biofilm and colonization in the lung [46]. We have not yet determined whether the growth conditions used in our study influence the amount of adherence, T2SS, and iron acquisition proteins or if enhanced secretion of any of these proteins occurred. We intend to explore these relationships in future studies. It is compelling that a simplified combination of planktonic and biofilm immunogens was amongst the most protective (Figure 5D,E). Perhaps this is not surprising, because an immunogen containing a greater array of antigens, may be predicted to stimulate greater protective immunity. However, more work will be needed to understand the positive and negative effects of adding and substituting immunogen preparations. The extent to which antigens are presented in specific types of infection is incompletely understood. Using different modes of growth to generate whole-cell vaccines is likely to present specific proteins that are crucial for the multiple stages of pathogenesis and result in layers of protection for the host. In addition, this is certainly not a species-specific phenomenon, and in fact, it is likely an important consideration for all bacterial vaccine approaches. In fact, supporting this idea, both planktonic and biofilm antigens were required for full protection with a *S*. *aureus* vaccine approach, where individually only ~50% protection was observed [47]. Another important consideration for immunogen optimization is cross-isolate protection. The demonstration of heterotypic protection will be critical for *A. baumannii* vaccine development and we are optimistic that methods discussed here will generate such an immunogen, which will be evaluated in our future studies.

Studies suggest that bacteria and viruses killed by gamma radiation are more immunogenic than those killed by chemicals such as formaldehyde [23,48]. The use of MDP during aqueous irradiations to further preserve immunogenicity while eliminating infectivity provides a novel approach for the production of whole-cell *A. baumannii* killed vaccines. Importantly, MDP did not protect *A. baumannii* from sterilization with 10 kGy of gamma radiation (Figure 3). Thus, whole *A. baumannii* vaccines could prospectively be developed against the most clinically relevant community-acquired and nosocomial *Acinetobacter* strains including *A. calcoaceticus*, *Acinetobacter* genomic species 3, and *Acinetobacter* genomic species 13TU, collectively referred to as *A. calcoaceticus–A. baumannii* complex [12]. The inactivated whole cell approach to vaccine production eliminates the time-consuming identification of protective subunit antigens and production of recombinant proteins. In addition, the relatively easy access to irradiation facilities make this approach rapid, safe, cost-effective, and readily scalable. Interestingly, in this study, no statistical difference in protection was seen in samples prepared with or without the MDP complex. In previous mouse challenge studies of MRSA and viruses [21], the inclusion of MDP during irradiation led to immunogens that were statistically more protective than analogs irradiated without MDP. The potential independence of MDP in the present study may be caused by the lower dose of radiation used to inactivate *A*. *baumannii* (i.e., 10 kGy) compared to doses used for other pathogens (i.e., 25 to 45 kGy). The reduced dose of radiation results in reduced concentrations of ROS. Additional studies will inform us as to the necessity of including the MDP complex during irradiation. Lower doses of immunogen may reveal differences in MDP +/− immunogens.

We preserved *A. baumannii* epitopes at gamma radiation doses safely above those needed to ensure sterilization of the aqueous preparations. On the other hand, the simplicity and economy of producing and screening these inactivated *A. baumannii* vaccine candidates could also serve to down-select protein immunogens discernable by 2-D gel and other analyses (Figure 2) to develop second-generation subunit vaccines.

Further optimization of strain, culture conditions, growth stage-dependent epitope presentation, and irradiation conditions, as well as administration of adjuvants, may further enhance the immunogenicity and efficacy of MDP-irradiated *A. baumannii* vaccine candidates. Testing in additional animal models for different clinical indications and studying the immunological correlates of protection will be critical for our understanding the similarities and differences between these different vaccine candidates. We believe these initial findings show that the MDP-irradiated vaccine approach represents a safe and rapidly adaptable vaccine strategy against emerging *A. baumannii* strains, and this knowledge will help to evaluate other whole-cell, killed strategies as well as identify candidates for recombinant protein vaccine approaches.

## 5. Conclusions

In conclusion, we demonstrate that whole-cell inactivated *A. baumannii*, cultured under specific conditions, can stimulate protective immunity in mice.

## Figures and Tables

**Table 1:** AB5075 growth conditions and sample designations.

Culture Designation	Growth Phase	Nutritional Source	Time of Harvest	Growth Platform
**P1**	Planktonic	TSB	Overnight	Agitated Broth
**P2**	Planktonic	TSB	2 h	Agitated Broth
**B1**	(Submerged) Biofilm	M9	2 days	Stationary Broth
**B2**	(Colony) Biofilm	M9	Overnight	Stationary Agar
**B3**	(Colony) Biofilm	TSA + SB	Overnight	Stationary Agar

TSB, tryptic soy broth; M9, M9 minimal media or M9 agar; TSA, trypticase soy agar; SB, sheep red blood. All cultures were grown at 37 °C under standard aerobic conditions.

**Table 2:** Table comparing protein sizes from cultures.

Culture Designation:	P1	P2	B1	B2	B3
		150	150	150	
		130	130		
		115	115		
**Unique/Distinct Bands kDa**:	80			80	80
		75	75		
		65			
		40	40		
	22				22

## Data Availability

No new data were created or analyzed in this study. Data sharing is not applicable to this article.

## Disclaimer

Material has been reviewed by the Walter Reed Army Institute of Research (WRAIR) and by the Uniformed Services University of the Health Sciences (USUHS). There is no objection to its presentation and/or publication. The opinions and assertions contained herein are the private ones of the authors and are not to be construed as official or reflecting the views of the Department of Defense, the Uniformed Services University of the Health Sciences, the Department of the Army, or any other agency of the U.S. Government.
